# The complications of irradiation or busulfan for CAYA before hematopoietic stem cell transplantation: a systematic review and meta-analysis

**DOI:** 10.3389/fped.2026.1865647

**Published:** 2026-09-16

**Authors:** Jiahe Zhang, Kaili Wei, Ziyan Du, Menglin Wu, Junqi Peng, Chun Chen, Jing Yang

**Affiliations:** 1 Zhongshan School of Medicine, Sun Yat-sen University, Shenzhen, China; 2 Pediatric Hematology Laboratory, Division of Hematology/Oncology, Department of Pediatrics, The Seventh Affiliated Hospital of Sun Yat-Sen University, Shenzhen, China

**Keywords:** busulfan, CAYA, complications, conditioning regimens, hematopoietic stem cell transplantation, total body irradiation

## Abstract

**Introduction:**

Hematopoietic stem cell transplantation (HSCT) represents a curative therapeutic modality for children, adolescents, and young-adult (CAYA) patients with various life-threatening hematological malignancies and non- malignant haematological disorders. Conditioning regimens, a cornerstone of HSCT, typically involve total body irradiation (TBI) or busulfan (Bu)-based myeloablation. However, the optimal conditioning strategy for CAYA populations remains controversial, and high-quality evidence regarding long-term complications is lacking. This systematic review and meta-analysis aimed to comprehensively compare the efficacy and safety profiles of TBI and Bu-based conditioning regimens in CAYA-HSCT recipients.

**Methods:**

We systematically searched PubMed, Embase, Cochrane Library, Web of Science, Scopus, ClinicalTrials.gov, Wanfang Data, and CNKI databases from inception to August 3, 2025. Eligible studies included prospective or retrospective cohort studies and randomized controlled trials involving CAYA patients (0–25 years) undergoing HSCT with TBI or Bu-based conditioning. Primary outcomes were overall survival (OS), event-free survival (EFS), transplant-related mortality (TRM), and relapse rate. Secondary outcomes included seven key long-term complications: growth retardation, thyroid dysfunction, hypogonadism, secondary tumors, cataracts, neurological complications, and chronic graft-vs.-host disease (cGVHD).

**Results:**

A total of 4,928 studies were identified, with 26 ultimately included in the meta-analysis. The results showed that Bu conferred survival benefits for patients with AML and cohorts comprising both malignant and non-malignant disease cases, whereas TBI yielded superior EFS and relapse-control outcomes in ALL patients. Five long-term complications (growth retardation, thyroid dysfunction, hypogonadism, secondary tumors and cataracts) occurred at significantly lower rates in the Bu group. Sex-related heterogeneity was observed for hypogonadism with higher Bu-associated risk in one female- only cohort. No significant differences were observed in neurological complications or cGVHD between the two groups.

**Discussion:**

In conclusion, conditioning-regimen selection should be individualized according to disease subtype. For ALL patients, clinicians need to balance the survival- and relapse-preventive advantages of TBI against its long-term toxic effects on children's growth, development and vital organs. For AML patients, the survival benefits of Bu-based conditioning should be weighed against sex- dependent toxic risks. Early preventive-interventional measures and adequate education for patients and guardians are essential for CAYA patients receiving TBI or Bu-based conditioning.

## Introduction

Hematopoietic stem cell transplantation (HSCT) has revolutionized treatment for children, adolescents, and young adults (CAYA) with hematological malignancies (e.g., acute lymphoblastic leukemia, acute myeloid leukemia) and non-malignant disorders (e.g., severe aplastic anemia, inherited metabolic diseases) (1, 2). The primary goal of pre-transplant conditioning is to eradicate malignant cells or dysfunctional hematopoietic tissue and induce immunosuppression to facilitate donor stem cell engraftment (3). Two main conditioning strategies have been widely adopted: total body irradiation (TBI) and busulfan (Bu)-based chemotherapy (4). Since the 1980s, fractionated TBI combined with cyclophosphamide (Cy) has been a standard conditioning regimen, leveraging its potent myeloablative and immunosuppressive effects (5). However, TBI is associated with significant acute and late toxicities, including organ damage, growth impairment, and secondary malignancies, which are particularly concerning in CAYA populations due to their immature organ systems and longer life expectancy (6, 7).

In contrast, Bu-based regimens emerged as a radiation-free alternative in the 1980s, with Bu/Cy demonstrating efficacy in acute myeloid leukemia (AML) (8). Subsequent modifications, such as dose adjustments and combination with other agents (e.g., fludarabine, thiotepa), have aimed to optimize efficacy while reducing toxicity (9, 10). Despite decades of clinical use, the optimal conditioning regimen for CAYA patients undergoing HSCT remains undefined. Previous meta-analyses have primarily focused on adult populations or mixed-age cohorts, with limited data specifically addressing CAYA-specific outcomes (11, 12). Children are not merely “small adults”—their developing organs, immune systems, and potential for long-term survival make them uniquely susceptible to conditioning-related toxicities (13). Key CAYA-specific concerns include growth and developmental disorders, endocrine dysfunction, and neurocognitive impairment, which can significantly impact quality of life (14). Several prior studies have compared TBI and Bu-based regimens, but results have been inconsistent. For example, a meta-analysis of AML patients suggested that Cy-TBI might be superior to Bu-Cy in survival outcomes, particularly in advanced disease (15), while another study reported higher Sinusoidal obstruction syndrome rates with Bu/Cy but lower late toxicities compared to TBI/Cy (16). However, these studies lacked sufficient power to assess CAYA-specific long-term complications. A recent pediatric-focused meta-analysis compared TBI and chemotherapy conditioning in acute lymphoblastic leukemia (ALL) but did not comprehensively evaluate several key long-term complications (17). To address these gaps, we conducted a systematic review and meta-analysis specifically focusing on CAYA patients undergoing HSCT with TBI or Bu-based conditioning. We aimed to (1) compare the efficacy of TBI and Bu in terms of overall survival (OS), event-free survival (EFS), transplant related mortality (TRM), and relapse rate; (2) assess the risk of seven key long-term complications; and (3) provide evidence-based recommendations for conditioning regimen selection in HSCT for CAYA populations.

## Materials and methods

### Search strategy and selection criteria

Literature screening was conducted from April 20, 2025, to August 3, 2025, in two phases: initial and secondary screening. During the initial screening, relevant studies were included based on a rapid review of abstracts. The secondary screening involved a detailed reading of the full texts to exclude studies with incomplete data, no full text available, or those that did not meet the criteria regarding interventions, study design, participants, and outcome measures. Five authors independently performed the screening process across various databases, covering both English and non-English journals, to ensure comprehensive literature inclusion.

Inclusion Criteria: (1) Population: CAYA (0–25 years old) undergoing allogeneic or autologous hematopoietic stem cell transplantation, regardless of the primary disease type (leukemia, bone marrow failure diseases, genetic metabolic diseases, etc.); (2) Intervention: Conditioning regimens including radiotherapy (total body irradiation, TBI, with a total dose of ≥12 Gy, fractionated or single-dose) or Bu (intravenous or oral formulation, regardless of dose intensity); (3) Comparison: Comparison between different conditioning regimens (e.g., TBI vs. Bu), which can also include sub-group comparisons of different doses/timing within the same regimen; (4) Outcomes: Studies must report at least one of the following complications: endocrine abnormalities (growth retardation, thyroid dysfunction, gonadal injury), neurological complications (epilepsy, leukoencephalopathy), secondary malignant tumors, chronic graft-vs.-host disease (cGVHD) or ocular toxicity (cataracts); (5) Study types: Prospective or retrospective cohort studies, randomized controlled trials (RCTs), and case-control studies (with a sample size of ≥10).

Exclusion Criteria: (1) Studies focusing on adults (≥26 years old) or mixed-age populations without separate reporting of CAYA data; (2) Studies using other chemotherapeutic agents (e.g., cyclophosphamide, fludarabine) without involving TBI or Bu; (3) Studies that do not clearly specify the components or doses of the conditioning regimens; (4) Non-primary research such as case reports, reviews, and conference abstracts; (5) Non-English literature without reliable translation; (6) Studies with duplicate data (the study with the longest follow-up time is retained); (7) Studies with a post-transplant follow-up period of <3 months (insufficient for evaluating medium- to long-term complications).

### Literature search

The aggregation of included literature was completed on September 3, 2025, followed by quality assessment and data extraction. Quality assessment was performed using the “Risk of Bias Table” from Review Manager 5.4.1 obtained from the Cochrane website (https://www.cochrane.org/), analyzing seven aspects: selection bias, performance bias, detection bias, attrition bias, reporting bias, and other biases. Data extraction involved compiling basic information and outcome data from the studies. Focusing on CAYA's health and development, we selected four primary outcomes (OS, EFS, TRM, and relapse rate) and seven secondary outcomes related to complications (growth retardation, thyroid dysfunction, hypogonadism, secondary tumors, cataracts, neurological complications and cGVHD). Other complications mentioned in the literature were also noted. To ensure data reliability and analyzability, for studies providing cumulative incidence rates (with 95% confidence intervals) of events and complications, we used R version 4.5.1 (https://cran.r-project.org/src/base/R-4) and the statistical methods described by Parmar in 1998 to calculate hazard ratios (HRs) with TBI as the reference standard for further analysis (14).

### Data extraction and statistical analysis

Outcome data were organized and completed on October 6, 2025, followed by data analysis. For the four primary outcomes, which were related to the number of events and time, we calculated overall HRs and 95% CIs using the inverse variance method based on the Bu/TBI HRs. For the secondary outcomes of complications, given the significant heterogeneity in follow-up times and that most studies reported event numbers, time factors were ignored, and we used the Mantel-Haenszel method to calculate odds ratios (ORs) and 95% CIs. For studies only providing cumulative incidence rates of complications, after data transformation to derive HRs, we calculated overall HRs and 95% CIs using the inverse variance method. Studies enrolling patients with non-malignant disorders were excluded from the analysis of relapse, while studies containing autologous hematopoietic stem cell transplantation recipients were excluded for cGVHD. To strengthen the robustness and interpretability of our analyses, subgroup analyses were performed for primary outcomes stratified by disease spectrum. And for secondary outcomes, subgroup stratifications were conducted according to transplant setting, disease spectrum, and median follow-up duration (classified as <5 years and ≥5 years). R version 4.5.1 was used for analyzing all primary and secondary outcome data. Considering the substantial clinical heterogeneity and the limited available data, a random-effects model was adopted for all analyses regardless of the results of heterogeneity testing.

## Results

A total of 4,928 studies were identified through the literature search, with 199 included in the initial screening, 30 in the secondary screening, and 26 finally eligible for meta-analysis. Additionally, four related studies were described and discussed separately (Figure 1). The literature quality assessment revealed that most included studies were retrospective, with only three being prospective. Measurement bias was low, but selection bias, reporting bias, and other biases were often unclear (Figures 2, 3). Based on systematic literature screening and data extraction, we selectively focused on several key clinical variables for subgroup stratification, including sex (male proportion), transplant setting, disease spectrum, and median follow-up duration. Transplant setting was categorized as mixed-population and allogeneic only. Disease spectrum was stratified into mixed haematological malignancies, mixed malignant and non-malignant diseases, and single specific disease subtypes. Variables including graft source (e.g., PBSC, BM), disease stage (e.g., CR1, CR2), and cytogenetic risk were not extracted or analyzed due to insufficient reporting, substantial data missingness, and limited availability across eligible studies. The basic information of the 26 studies included in the meta-analysis is summarized in Table 1, while the four studies analyzed separately are summarized in Table 2. Patient characteristics and outcome information comparing the busulfan-based (Bu) and total-body-irradiation-based (TBI) groups across the 26 studies included in this meta-analysis are summarized as well (Supplementary Table 1). Although patients in the TBI group tended to be older than those in the Bu group, and several studies showed imbalanced disease distributions (ALL predominated in the TBI arm, while AML or non-malignant disorders were over-represented in the Bu arm), baseline data suggested acceptable comparability between the two groups.

**Figure 1:**
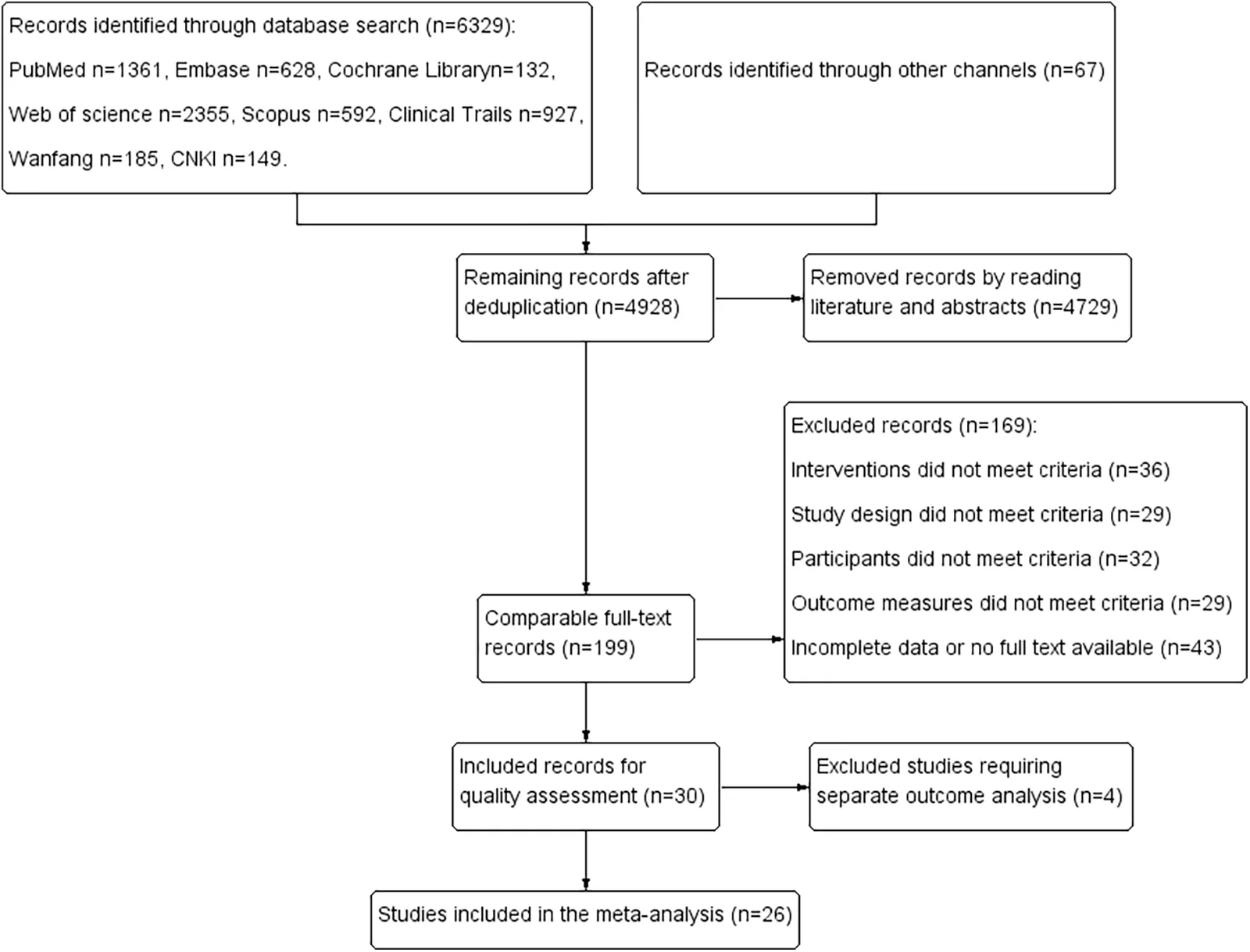
Flow diagram of study inclusion for meta-analysis. *Reviewers independently screened the retrieved literature initially by titles and abstracts, followed by secondary full-text assessment for potentially eligible studies. Screening results were cross-compared, and any disagreements were resolved through discussion; a third reviewer was involved for arbitration if consensus could not be reached.

**Figure 2:**
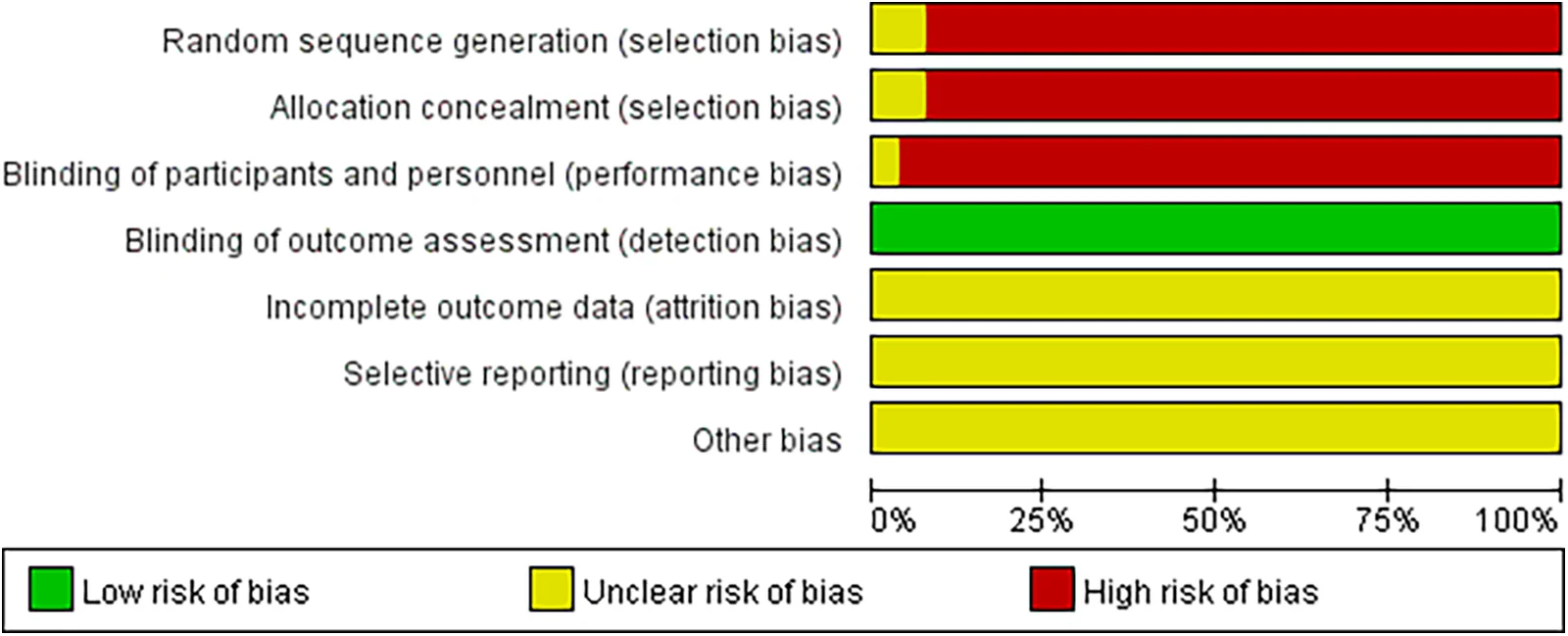
Risk of bias graph: review authors' judgements about each risk of bias item presented as percentages across all included studies. *Risk-of-bias assessment was performed according to the Cochrane risk-of-bias tool. Each study was evaluated across the following domains: random sequence generation, allocation concealment, blinding of participants and personnel, blinding of outcome assessment, incomplete outcome data, selective reporting, and other sources of bias. Judgements for each domain were classified as low, unclear or high risk. Bars represent the proportion of studies judged to be at low, unclear or high risk of bias within each domain.

**Figure 3:**
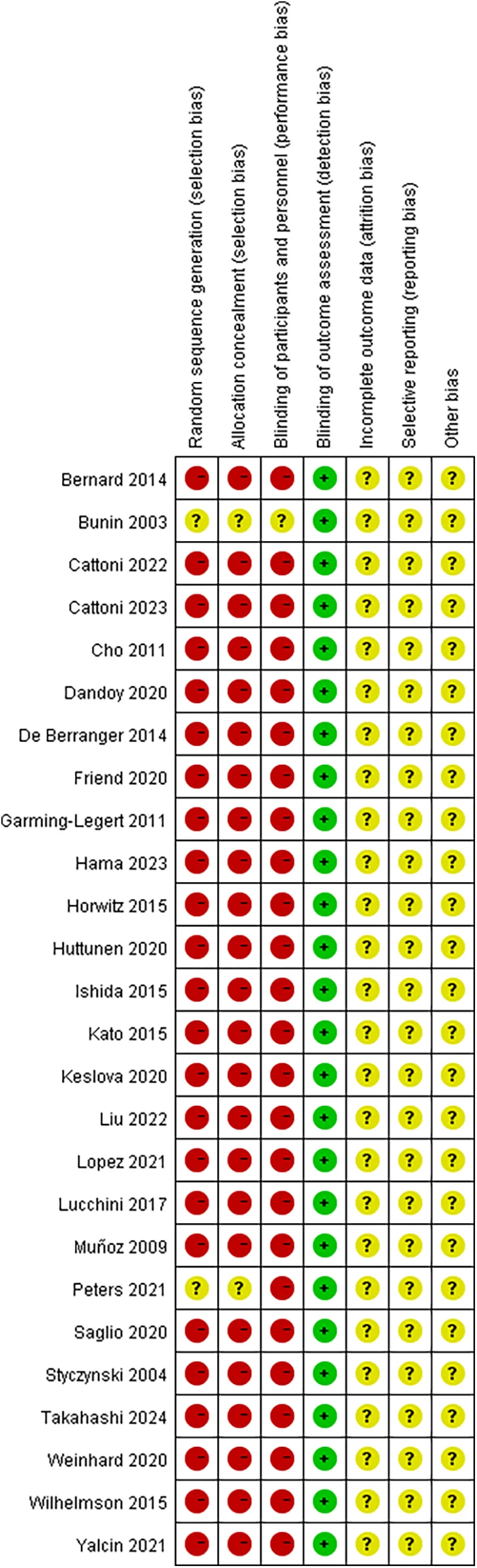
Risk of bias summary: review authors' judgements about each risk of bias item for each included study. *The Cochrane risk-of-bias tool was used to assess methodological quality. Each row corresponds to one included study, and each column represents a bias domain: random sequence generation, allocation concealment, blinding of participants and personnel, blinding of outcome assessment, incomplete outcome data, selective reporting, and other bias. Green indicates low risk of bias; yellow indicates unclear risk of bias; red indicates high risk of bias.

**Table 1:** Characteristics of included studies and patients (comprehensively analyzed).

Study (Author, year)	Study design	No. of patients (TBI + Bu/total)	Age (yrs) (median age, age range)	Gender (male%)	Transplant setting	Disease spectrum	Median follow-up (yrs)
Bernard et al. (15)	Retrospective cohort study, multicenter	240/240	7.9 (0.5–20.3)	63.8	Mixed-population	Mixed haematological malignancies	12.1
Bunin et al. (16)	Randomized controlled trial, multicenter	42/42	8.0 (0.5–20.0)	61.9	Allogeneic	ALL	3.6
Cattoni et al. (17)	Retrospective observational study, single-center	198/244	9.4 (0–21.0)	65.0	Allogeneic	Malignant and non-malignant mixed	9.7
Cattoni et al. (18)	Retrospective observational study, single-center	93/130	9.0 (0–18.0)	100	Allogeneic	Malignant and non-malignant mixed	> 5
Cho et al. (19)	Retrospective observational study, single-center	42/50	9.9 (0–18.0)	0	Mixed-population	Malignant and non-malignant mixed	> 5
Dandoy et al. (48)	Retrospective cohort study, multicenter	624/624	NA (0–21.0)	51.0	Allogeneic	AML	4.2
De Berranger et al. (20)	Retrospective cohort study, multicenter	226/226	10.0 (0.5–18.2)	46.9	Allogeneic	AML	4.9
Friend et al. (21)	Retrospective cohort study, single-center	57/57	11.0 (1.0–25.0)	49.0	Allogeneic	ALL	2.5
Garming-Legert et al. (22)	Retrospective cohort study, single-center	74/74	NA (0.5–13.0)	52.7	Allogeneic	Malignant and non-malignant mixed	8.0
Hama et al. (23)	Retrospective observational study, multicenter	177/203	2.0 (0–16.0)	46.8	Mixed-population	AML	8.9
Horwitz et al. (24)	Prospective cohort study, multicenter	271/271	8.7 (0–18.0)	62.0	Mixed-population	Mixed haematological malignancies	10.3
Huttunen et al. (25)	Retrospective cohort study, single-center	50/50	NA (0.6–17.1)	62.0	Allogeneic	Malignant and non-malignant mixed	2.0
Ishida et al. (26)	Retrospective cohort study, multicenter	220/220	9.0 (0–17.0)	49.0	Allogeneic	AML	2.3
Kato et al. (27)	Retrospective observational study, multicenter	118/175	0.75 (0–2.0)	NA	Allogeneic	ALL	4.9
Keslova et al. (28)	Retrospective observational study, single-center	321/426	7.9 (0.1–20.5)	63.9	Allogeneic	Malignant and non-malignant mixed	9.9
Liu et al. (29)	Retrospective cohort study, single-center	311/311	11.9 (0.6–25.2)	56.9	Allogeneic	Mixed haematological malignancies	6.2
Lopez et al. (30)	Retrospective cohort study, multicenter	231/255	11.4 (0–18.0)	100	Mixed-population	Mixed haematological malignancies	16.5
Lucchini et al. (31)	Retrospective cohort study, multicenter	498/631	11.9 (2.0–18.0)	54.2	Allogeneic	AML	4.6
Muñoz et al. (32)	Retrospective observational study, multicenter	24/24	10.0 (3.0–18.0)	45.8	Allogeneic	MDS	0.3
Peters et al. (33)	Randomized controlled trial, multicenter	303/413	10.4 (4.0–21.0)	65.0	Allogeneic	ALL	2.1
Saglio et al. (35)	Retrospective observational study, multicenter	670/670	9.0 (3.0–18.0)	62.0	Allogeneic	Mixed haematological malignancies	7.0
Styczynski et al. (36)	Retrospective observational study, single-center	20/29	9.0 (0.5–20.0)	59.0	Allogeneic	Malignant and non-malignant mixed	0.9
Takahashi et al. (37)	Retrospective study, multicenter	550/550	NA (0–21.0)	NA	Allogeneic	AML	4.9
Weinhard et al. (38)	Retrospective cohort study, single-center	74/74	7.0 (0–13.0)	62.2	Allogeneic	Malignant and non-malignant mixed	> 5
Wilhelmson et al. (39)	Retrospective cohort study, multicenter	175/204	8.0 (0.4–19.0)	54.0	Allogeneic	Malignant and non-malignant mixed	12.0
Yalcin et al. (40)	Retrospective cohort study, single-center	72/72	8.5 (0.8–17.4)	65.3	Allogeneic	ALL	2.1

Bu, busulfan; TBI, total body irradiation; NA, not available; ALL, acute lymphoblastic leukemia; AML, acute myeloid leukemia; MDS, myelodysplastic syndromes. No. of patients (TBI + Bu/Total): patients receiving TBI or Bu-based conditioning/total study-enrolled patients; only myeloablative conditioning patients were eligible. Detailed conditioning parameters were not analyzed due to methodological heterogeneity. Age: age at transplantation; mean age substituted missing median age, with some studies lacking age data. Mixed-population: studies enrolling both allogeneic and autologous transplant recipients, in which the majority of patients were allogeneic transplant cases.

**Table 2:** Characteristics of included studies and patients (independently analyzed).

Study (Author, year)	Study design	Conditioning regimen	No. of patients	Age (yrs) (median age, age range)	Sex (male%)	Disease stage	Donor type	Graft source	Median follow-up
Kahn et al. (42)	Retrospective cohort study	TBI/Cy vs. Bu/Cy	3,721	5.5 (0–21)	60.5	NA	NA	BM 56.8%	>7 years
								PBSC 13.3%	
								Cord 30%	
Mejdahl Nielsen et al. (43)	Cross-sectional study	TBI vs. non-TBI	98	NA (0–16)	100	NA	Allo	HSC	18.3 years
Rayner et al. (34)	Retrospective cohort study, single-center	Bu vs. TBI	49	7.6 (0.15–22.9)	63	NA	MRD 2%	HSC	>100 days
							MSD 51%		
							URD 47%		
Vatanen et al. (44)	Retrospective study, two-centers	fTBI vs. Bu vs. Cy vs. Cy/TLI	92	9 (1–19)	0	CR1	RD 65%	HSC	13 years
						CR2	URD 35%		

Bu, busulfan; TBI, total body irradiation; fTBI, fractional total body irradiation; TLI, total lymphoid irradiation; Cy, cyclophosphamide; CR, complete remission; Allo, allogeneic; MRD, matched related donor; MSD, matched sibling donor; MUD, matched unrelated donor; URD, unrelated donor; RD, related donor; BM, bone marrow; PBSC, peripheral blood stem cell; HSC, hematopoietic stem cells; NA, not available.

### Primary outcomes

The HR analysis for the four primary outcomes showed no significant differences between Bu and TBI groups (*p* > 0.05). Among these four primary outcomes, only TRM exhibited good homogeneity across studies (*I*^2^ < 40%, *p* > 0.05) (Figures 4A–D). Funnel plots (Figures 4E–H) and Egger's linear regression tests of funnel-plot asymmetry (Supplementary Material) suggested low likelihood of potential publication bias (*p* > 0.05). To further explore the stratified efficacy differences between regimens and potential sources of heterogeneity, subgroup analyses were performed based on the study disease spectrum. For OS and EFS, significant inter-study heterogeneity was detected (*p* < 0.05) (Figures 5A,B). Subgroup results for OS demonstrated that the Bu regimen yielded significantly lower mortality risk than the TBI regimen in patients with AML and in populations with malignant and non-malignant diseases [OR: 0.82 (95%CI: 0.68–0.98, *P* < 0.05) and OR: 0.67 (95%CI: 0.45–0.99, *P* < 0.05), respectively], indicating superior survival benefits of Bu in these subgroups (Figure 5A). Conversely, for EFS, in the ALL subgroup, Bu was associated with a significantly higher event risk compared with TBI [OR: 2.18 (95%CI: 1.50–3.18, *P* < 0.05)] (Figure 5B). For TRM, subgroup analyses showed good inter-study homogeneity (*p* > 0.05), and Bu significantly reduced the TRM risk relative to TBI in AML patients [0.75 (95%CI: 0.57–0.98, *P* < 0.05)] (Figure 5C). For relapse, the ALL subgroup exhibited favorable homogeneity (*p* > 0.05), with patients receiving Bu experiencing a significantly higher relapse risk than those treated with TBI [2.42 (95%CI: 1.59–3.67, *P* < 0.05)] (Figure 5D).

**Figure 4:**
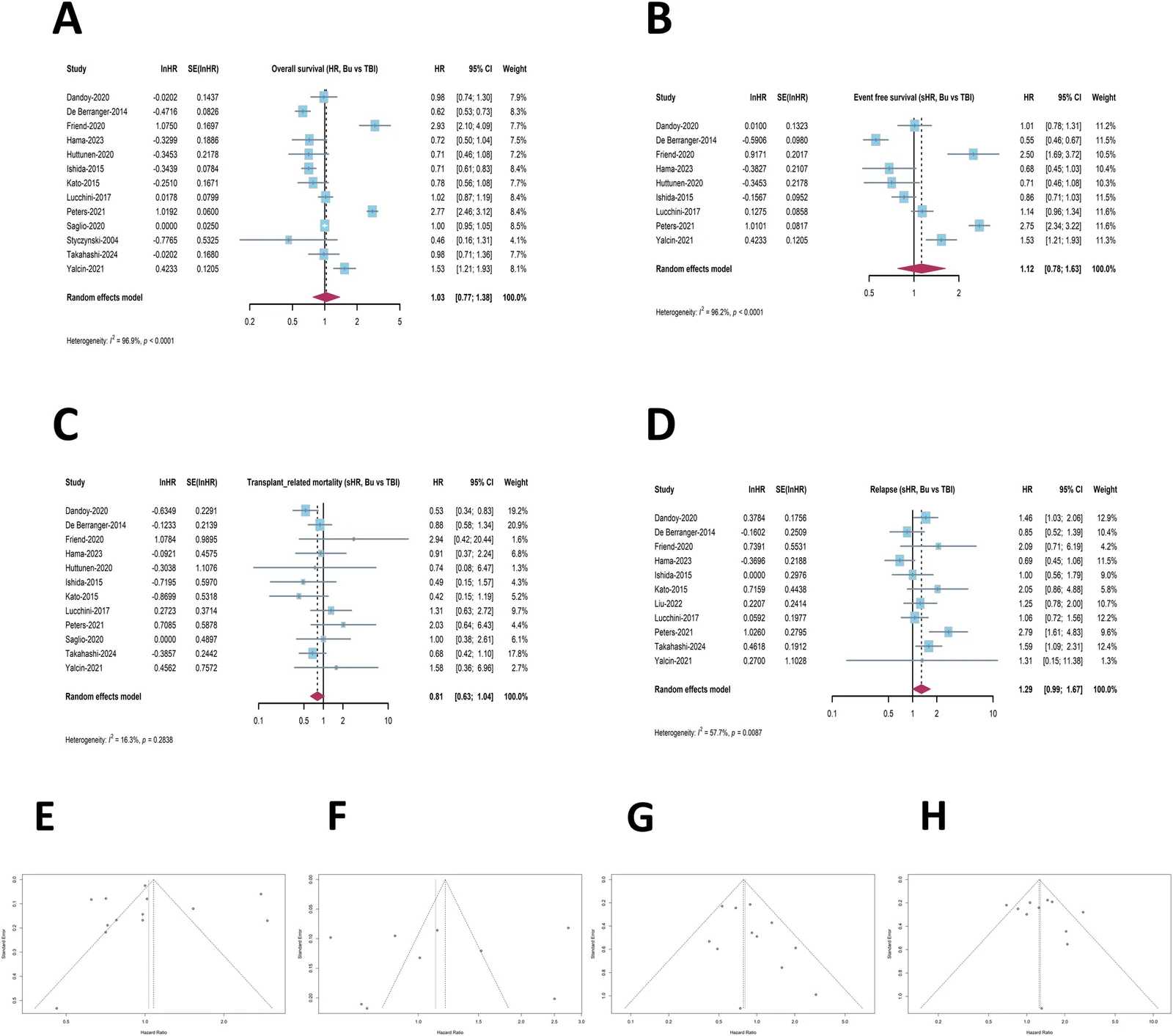
Primary outcomes of patients included in the meta-analysis (forest plots & funnel plots). *(A) Forest plot for OS (HR, Bu vs. TBI). The HR for Bu vs. TBI was 1.03 (95%CI: 0.77–1.38, *P* > 0.05, heterogeneity: *P* < 0.05). **(B)** Forest plot for EFS (HR, Bu vs. TBI). The HR for Bu versus TBI was 1.12 (95%CI: 0.78–1.63, *P* > 0.05, heterogeneity: *P* < 0.05). **(C)** Forest plot for TRM (HR, Bu vs. TBI). The HR for Bu vs. TBI was 0.81 (95%CI: 0.63–1.04, *P* > 0.05, heterogeneity: *P* > 0.05). **(D)** Forest plot for relapse (HR, Bu vs. TBI). The HR for Bu vs. TBI was 1.29 (95%CI: 0.99–1.67, *P* > 0.05, heterogeneity: *P* < 0.05). **(E)** Funnel plot for OS. **(F)** Funnel plot for EFS. **(G)** Funnel plot for TRM. **(H)** Funnel plot for relapse. All meta-analyses were performed under random-effects models. HR > 1 indicates higher risk in the Bu group; HR < 1 indicates higher risk in the TBI group.

**Figure 5:**
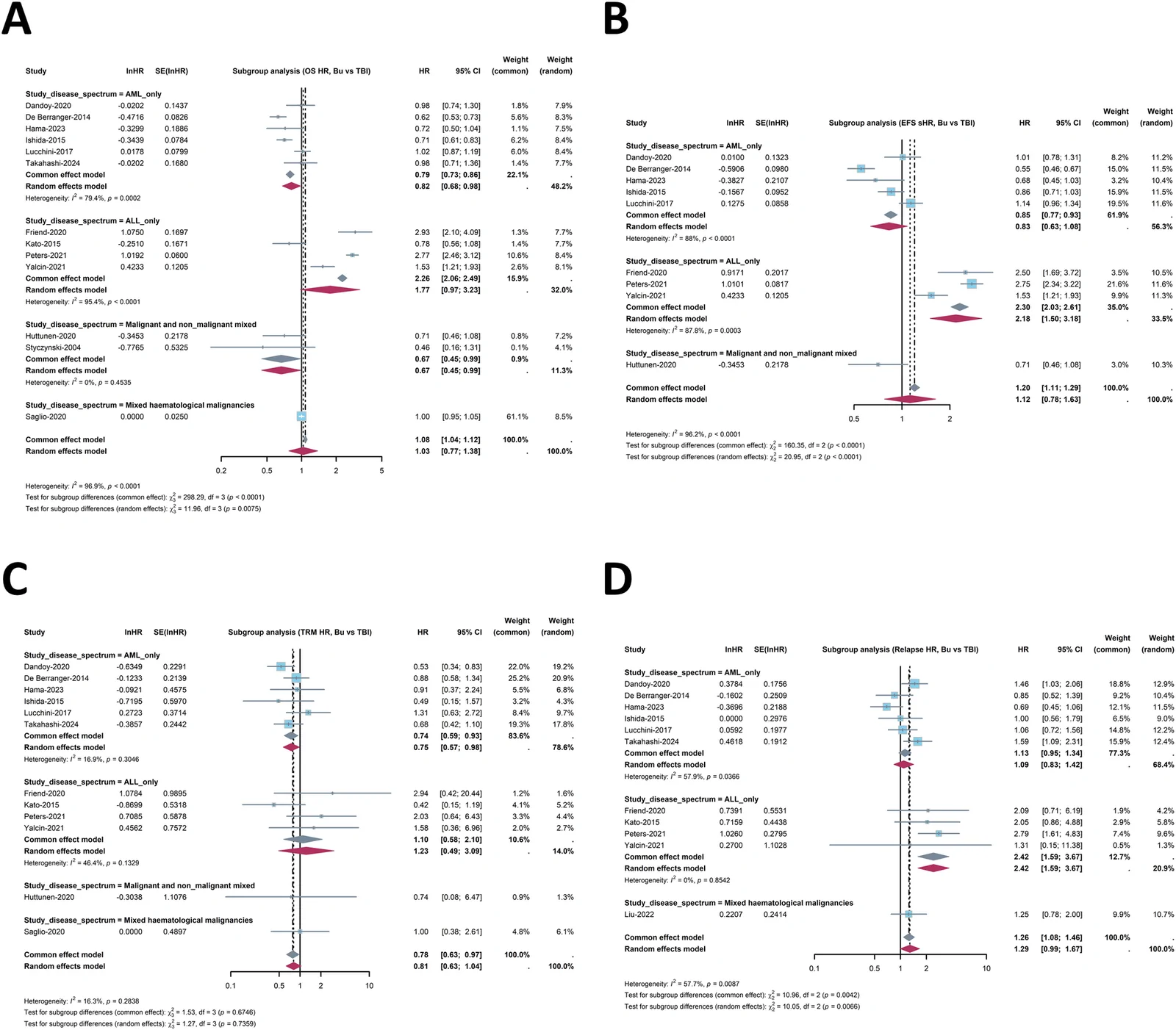
Subgroup analyses of primary outcomes. *(A) Subgroup meta-analysis for OS. In AML patients, the HR for Bu vs. TBI was 0.82 (95%CI: 0.68–0.98, *P* < 0.05, heterogeneity: *P* < 0.05); in ALL patients, the HR for Bu vs. TBI was 1.77 (95%CI: 0.97–3.23, *P* > 0.05, heterogeneity: *P* < 0.05); in malignant and non-malignant patients, the HR for Bu vs. TBI was 0.67 (95%CI: 0.45–0.99, *P* < 0.05, heterogeneity: *P* > 0.05). **(B)** Subgroup meta-analysis for EFS. In AML patients, the HR for Bu vs. TBI was 0.83 (95%CI: 0.63–1.08, *P* > 0.05, heterogeneity: *P* < 0.05); in ALL patients, the HR for Bu vs. TBI was 2.18 (95%CI: 1.50–3.18, *P* < 0.05, heterogeneity: *P* < 0.05). **(C)** Subgroup meta-analysis for TRM. In AML patients, the HR for Bu vs. TBI was 0.75 (95%CI: 0.57–0.98, *P* < 0.05, heterogeneity: *P* > 0.05); in ALL patients, the HR for Bu vs. TBI was 1.23 (95%CI: 0.49–3.09, *P* > 0.05, heterogeneity: *P* > 0.05). **(D)** Subgroup meta-analysis for relapse. In AML patients, the HR for Bu vs. TBI was 1.09 (95%CI: 0.83–1.42, *P* > 0.05, heterogeneity: *P* < 0.05); in ALL patients, the HR for Bu vs. TBI was 2.42 (95%CI: 1.59–3.67, *P* < 0.05, heterogeneity: *P* > 0.05). Stratification was performed according to disease spectrum. Tests for subgroup interaction were reported. Random-effects models were applied.

### Secondary outcomes (long-term complications)

The seven complication outcomes varied in data volume, with some analyses including over 1,000 patients and others fewer than 100 (Figures 6, 7). Funnel plots suggested low likelihood of potential publication bias (Supplementary Figure 2). Five complications (growth retardation, thyroid dysfunction, hypogonadism, secondary tumors, and cataracts) showed significant differences (*p* < 0.05) (Figures 6A,B,E–G, 7A), while the other two (neurological complications and cGVHD) did not (*p* > 0.05) (Figures 6C,D,H). Except for hypogonadism (*p* < 0.05) (Figure 7A), the homogeneity of the remaining results was good. To further explore the sources of heterogeneity for hypogonadism, we performed subgroup analysis stratified by patient sex. Sex-stratified grouping substantially improved within-subgroup homogeneity (Figure 7B). For the incidence of hypogonadism, the Bu regimen was associated with a significantly lower risk than TBI in male-only studies [OR: 0.27 (95%CI: 0.16–0.46, *p* < 0.05)], as well as in mixed male and female studies [0.53 (95%CI: 0.32–0.87, *p* < 0.05)]. In the single female-only study, however, Bu yielded a significantly higher risk of hypogonadism than TBI (Figure 7B). For cGVHD (OR), subgroup analysis was conducted according to median follow-up duration. Subgroups with a follow-up of more than five years exhibited better homogeneity than those with shorter follow-up (<5 years) (Figure 7C). The protective trend of Bu against cGVHD relative to TBI declined in subgroups with a follow-up duration of more than 5 years compared with those with a follow-up of less than 5 years, although this change did not reach statistical significance, as confirmed by meta-regression analysis (Supplementary Material).

**Figure 6:**
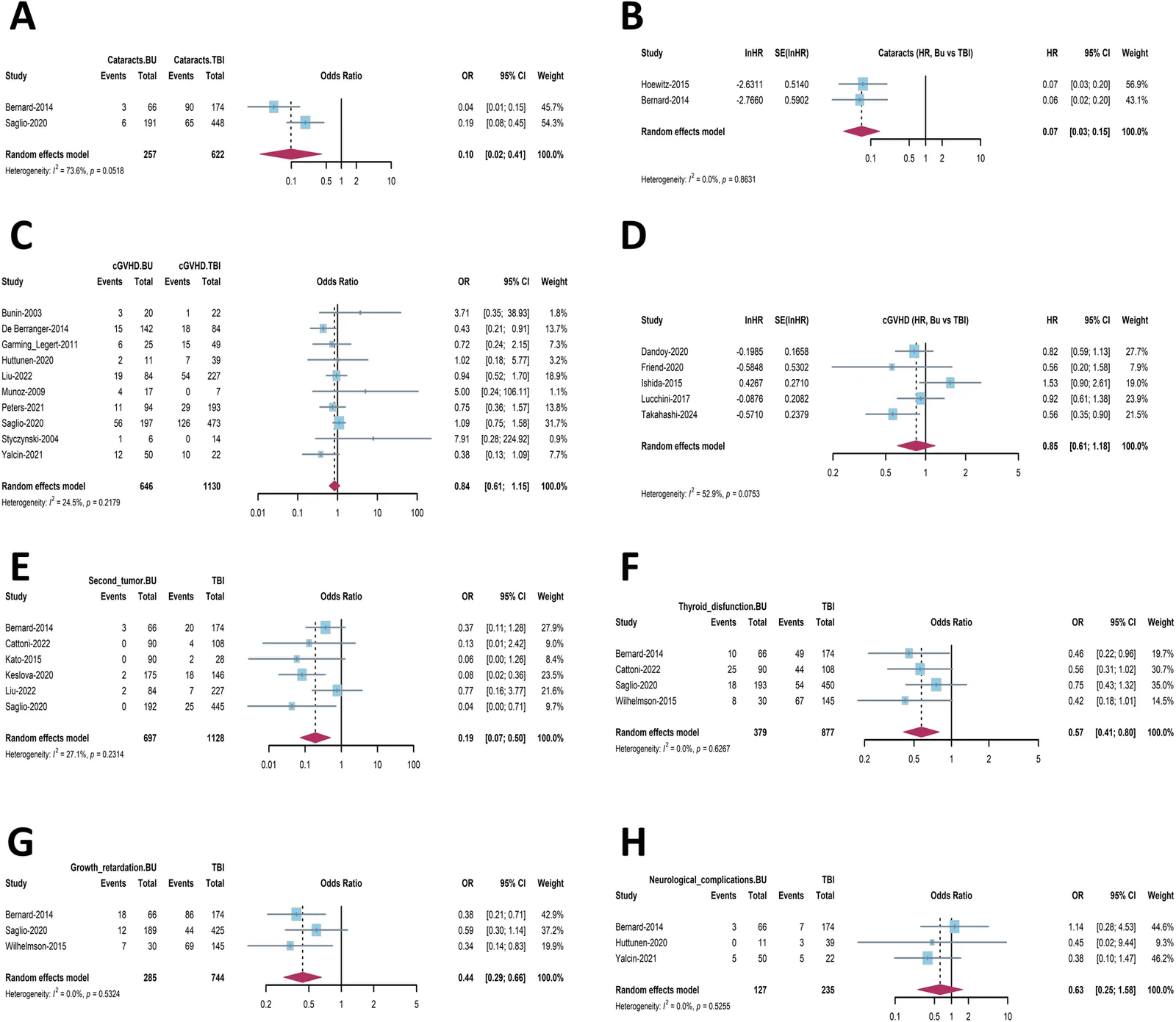
Secondary outcomes of patients included in the meta-analysis (forest plots). *(A) Forest plot of OR for cataracts. The OR (Bu vs. TBI) was 0.10 (95%CI: 0.02–0.41, *p* < 0.05, heterogeneity: *P* > 0.05). **(B)** Forest plot of HR for cataracts. The HR (Bu vs. TBI) was 0.07 (95%CI: 0.03–0.15, *p* < 0.05, heterogeneity: *P* > 0.05). **(C)** Forest plot of OR for cGVHD. The OR (Bu vs. TBI) was 0.84 (95%CI: 0.61–1.15, *p* > 0.05, heterogeneity: *P* > 0.05). **(D)** Forest plot of HR for cGVHD. The HR (Bu vs. TBI) was 0.85 (95%CI: 0.61–1.18, *p* > 0.05, heterogeneity: *P* > 0.05). **(E)** Forest plot of OR for second tumor. The OR (Bu vs. TBI) was 0.19 (95%CI: 0.07–0.50, *p* < 0.05, heterogeneity: *P* > 0.05). **(F)** Forest plot of OR for thyroid dysfunction. The OR (Bu vs. TBI) was 0.57 (95%CI: 0.41–0.80, *p* < 0.05, heterogeneity: *P* > 0.05). **(G)** Forest plot of OR for growth retardation. The OR (Bu vs. TBI) was 0.44 (95%CI: 0.29–0.66, *p* < 0.05, heterogeneity: *P* > 0.05). **(H)** Forest plot of OR for neurological complications. The OR (Bu vs. TBI) was 0.63 (95%CI: 0.25–1.58, *p* > 0.05, heterogeneity: *P* > 0.05). Random-effects models were used. OR < 1 or HR < 1 suggests lower complication risk in the Bu-conditioning arm.

**Figure 7:**
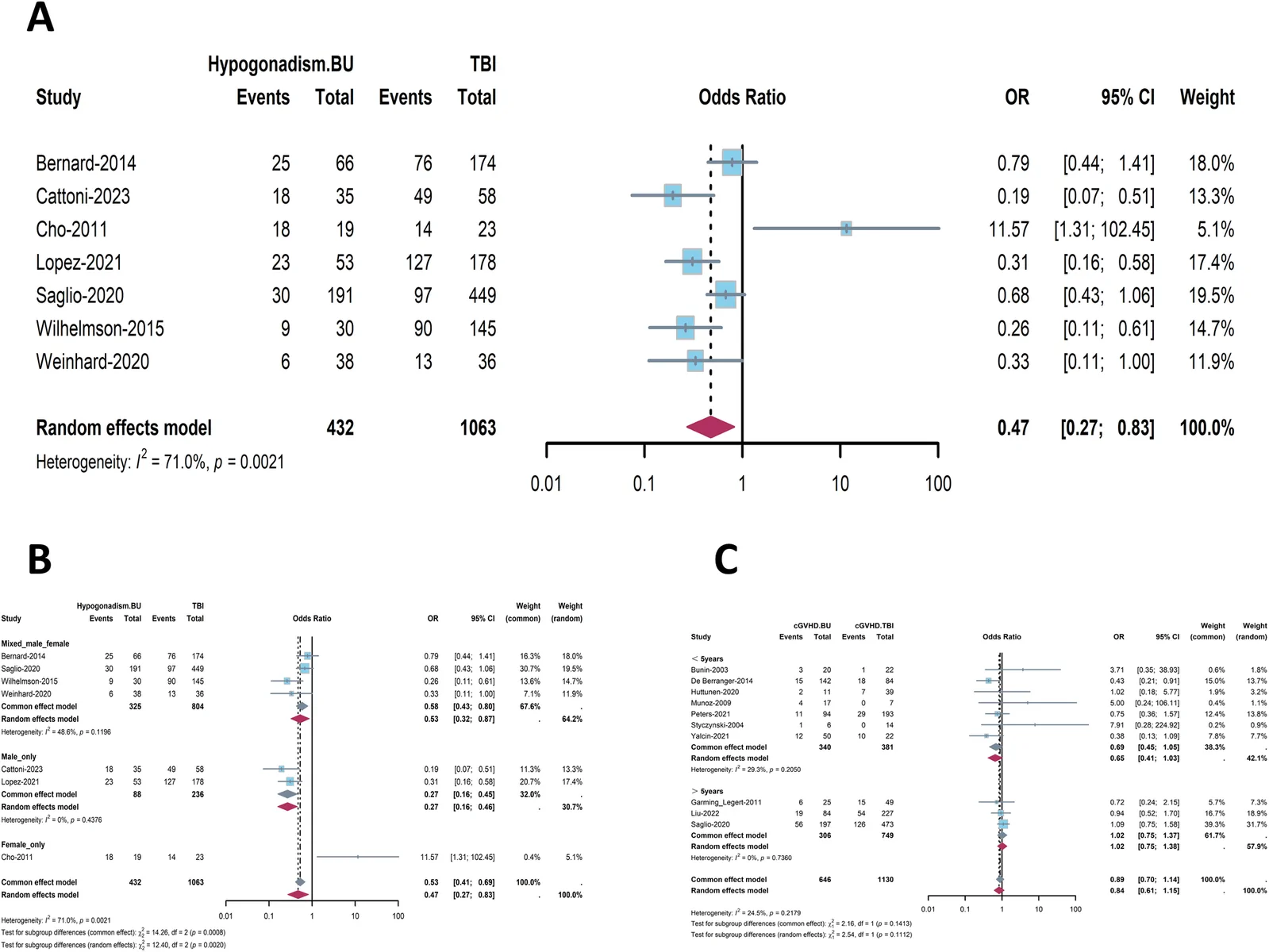
Secondary outcome (forest plot of OR for hypogonadism) and subgroup analyses of secondary outcomes (hypogonadism, cGVHD) of patients included in the meta-analysis. *(A) Forest plot of OR for hypogonadism. The OR (Bu vs. TBI) was 0.47 (95%CI: 0.27–0.83, *p* < 0.05, heterogeneity: *P* < 0.05). **(B)** Subgroup analysis of hypogonadism stratified by sex. In mixed population, the OR (Bu vs. TBI) was 0.53 (95%CI: 0.32–0.87, *p* < 0.05, heterogeneity: *P* > 0.05); in male patients, the OR (Bu vs. TBI) was 0.27 (95%CI: 0.16–0.46, *p* < 0.05, heterogeneity: *P* > 0.05); in female patients, the OR (Bu vs. TBI) was 11.57 (95%CI: 1.31–102.45, *p* < 0.05). **(C)** Subgroup analysis of cGVHD stratified by the median follow-up. In patients with a median follow-up of less than 5 years, the OR (Bu vs. TBI) was 0.65 (95%CI: 0.41–1.03, *p* > 0.05, heterogeneity: *P* > 0.05); in patients with a median follow-up of more than 5 years, the OR (Bu vs. TBI) was 1.02 (95%CI: 0.75–1.38, *p* > 0.05, heterogeneity: *P* > 0.05). Random-effects meta-analyses were conducted.

## Discussion

Sensitivity and subgroup analyses were not performed for cataracts owing to the limited number of eligible studies. Leave-one-out sensitivity analyses demonstrated robustness of the overall meta-analytic findings, for both primary outcomes (Supplementary Figure 1) and long-term post-transplant complications (Figure 8). Given that data for long-term endpoints including cataracts, growth retardation, thyroid dysfunction and hypogonadism were exclusively derived from studies with a median follow-up >5 years, whereas cGVHD (HR) data were all obtained from studies with a median follow-up <5 years, subgroup analyses stratified by median follow-up were not conducted for these outcomes. For the remaining long-term-complication endpoints, subgroup analyses were performed according to median follow-up, study transplant setting, and study disease spectrum. Nevertheless, additional subgroup analyses for long-term complications did not reveal improved homogeneity or other informative significant findings (Supplementary Figures 3, 4).

**Figure 8:**
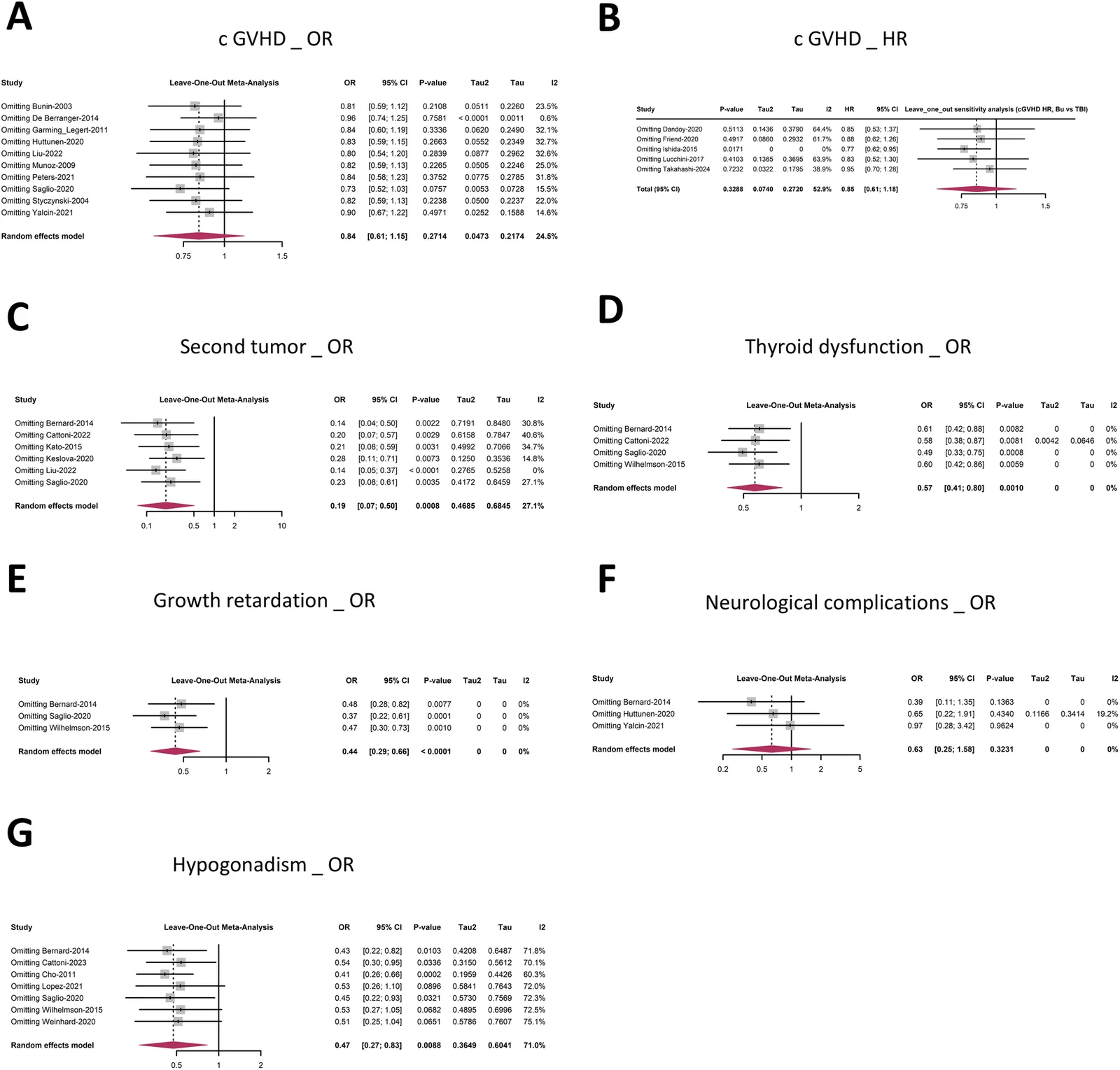
Leave-one-out sensitivity analyses for late complication endpoints. *(A) Leave-one-out sensitivity analysis for cGVHD (OR). **(B)** Leave-one-out sensitivity analysis for cGVHD (HR). **(C)** Leave-one-out sensitivity analysis for second tumour (OR). **(D)** Leave-one-out sensitivity analysis for thyroid dysfunction (OR). **(E)** Leave-one-out sensitivity analysis for growth retardation (OR). **(F)** Leave-one-out sensitivity analysis for neurological complications (OR). **(G)** Leave-one-out sensitivity analysis for hypogonadism (OR). Each study was sequentially omitted to evaluate the robustness of pooled effect estimates.

Our meta-analysis reveals that in CAYA patients undergoing HSCT, the differences in OS, EFS, and relapse rates between Bu and TBI conditioning regimens are not statistically significant. Nevertheless, stratified effects were identified across distinct disease subgroups: Bu yielded survival benefits in patients with AML and in cohorts containing both malignant and non-malignant disease cases, whereas TBI was more favorable for ALL patients in terms of EFS and relapse risk. Regarding long-term complications, Bu was associated with reduced risks of growth retardation, thyroid dysfunction, hypogonadism, secondary tumors, and cataracts in the pooled analysis. Sex-related heterogeneity was prominent for hypogonadism, where the protective effect of Bu was reversed in the single female-only study. While not statistically significant, TBI is also linked to higher rates of neurological complications and cGVHD.

The analysis included studies from 2003 to 2024, with most published post-2010. Our comprehensive search across major and niche databases ensures relatively updated and extensive results. While our analysis is CAYA-specific, its results align with several adult meta-analyses (10, 11). Compared to a prior pediatric meta-analysis, we offer a more in-depth analysis of complications (13). Below is a detailed interpretation of the findings:

In CAYA patients (<25 years old) with predominantly aggressive diseases, myeloablative conditioning is crucial for eradicating malignant cells and enhancing allogenic HSCT efficacy through the graft-vs.-tumor effect (3). TBI's ability to more comprehensively eliminate malignant cells compared to Bu, which relies on systemic circulation and localized permeation, may explain the better OS and lower relapse rates observed in TBI groups for ALL patients. However, TBI's long-term genomic instability poses greater irreversible risks, particularly for children whose growth, neurological development, and immune systems are highly vulnerable. While Bu was historically linked to higher Sinusoidal obstruction syndrome rates (7), advancements in prophylactic therapies have mitigated this risk, aligning Bu and TBI outcomes in contemporary studies.

Moreover, bone marrow hematopoietic dysfunction is a notable toxicity of pretransplant conditioning. Kahn et al. observed an inverse relationship between patient age and the incidence of this dysfunction (42). Compared to the Bu group, patients receiving <400 cGy of TBI had half the risk, while those with ≥400 cGy had double the risk, and those with ≥800 cGy had 8.7 times the risk of the Bu group (42).

Sensitivity analysis for hypogonadism excluding Cho et al. yielded a significant result (*P* < 0.001, OR = 0.41) (Figure 8G), indicating a lower risk in the Bu group, which was consistent with the findings from subgroup analysis (Figure 7B). Cho's study population comprised only female patients, with higher bone age in the TBI group (*p* < 0.05), potentially introducing selection bias (19). Conversely, a male-only study reported significant hypogonadism in the TBI group (43). Notably, female patients, especially aged 10–20, are more prone to hypogonadism (35, 38, 42). Vatanen et al. highlighted cyclophosphamide's comparatively lower reproductive toxicity (44). Accordingly, sex-related differences should be taken into account when selecting conditioning regimens and implementing preventive strategies for gonadal-related complications.

In sensitivity analysis, data on secondary tumors from the Liu (29) study may attenuate the overall protective trend of the Bu regimen seen in the pooled analysis (Figure 8C). This finding could be attributable to patient-level disease heterogeneity (i.e., a higher proportion of AML and absence of ALL cases in the Bu group vs. predominant ALL cases in the TBI group) or other confounding factors including discrepancies in cytogenetic risk stratification (29).

Age's impact on outcomes is inconsistent across studies. Rayner et al. noted higher Sinusoidal obstruction syndrome incidence in younger patients (34), while another study found no significant interaction between donor type, age, and transplantation outcomes (41). Notably, TBI groups often include older patients than Bu groups (19, 21, 26, 40), possibly because very young patients are deemed unsuitable for TBI, highlighting the need for individualized treatment approaches alongside guideline adherence.

Although the influence of disease type on clinical outcomes was explored via subgroup analyses in our study, the relationship between disease severity and clinical prognosis remains to be further elucidated by well-designed prospective studies and additional systematic reviews. Saglio et al. reported a higher incidence of cataracts in patients with ALL compared with those with AML (35). Weinhard et al. observed a higher proportion of ALL cases within TBI groups (38), which aligns with TBI being regarded as a gold-standard conditioning regimen for ALL (6). Nevertheless, such imbalanced disease distribution across conditioning groups may confound the true association between patient prognosis and underlying disease type or severity. Some studies included only ALL patients (16, 21, 27, 33, 40), some only AML patients (20, 26, 31, 37), and one only MDS patients (32), while others encompassed various malignant and benign diseases. All these issues warrant further investigation.

Despite this, our analysis offers several insights for future research directions. Regarding graft sources, autologous transplants lack graft-vs.-tumor activity, but umbilical cord blood transplantation (UCBT), with its lower HLA matching requirements, shows promise and may bring hope to more patients (1). However, whether UCBT prognosis is linked to pre-transplant conditioning requires further clinical and systematic review studies. In conditioning regimens, antibody-based approaches could be the next major advancement in HSCT, necessitating larger clinical trials of antibody-related drugs and updated systematic reviews of conditioning regimens (45). The introduction of CAR-T cell therapy also calls for clinical studies and meta-analyses of its toxicities to determine its indications, contraindications, and potential to replace HSCT (46). While answers to these discussions remain elusive, it is expected that, like the Flu/Bu/TBI400 conditioning regimen for allogeneic HSCT in acute lymphocytic leukemia patients with poor performance status (47), more prospective randomized controlled trials will drive the updating of conditioning regimens. This will provide suitable transplant opportunities for more patients, extend their survival, and enhance their quality of life.

### Study limitations

Heterogeneity across included studies: Limitations arise from the retrospective nature of most included studies and their inherent heterogeneity. The enrolled studies varied in factors such as patient age stratification, cytogenetic risks, specific dosages of TBI/Bu, and graft sources. The heterogeneity of patients' living environments, medical care, and transplantation/recovery conditions remains unaccounted for. Significant disparities in sample sizes between TBI and Bu groups across different complication analyses suggest that more extensive research is needed. These differences may have introduced heterogeneity, which could impact the stability and generalizability of the pooled results.

Potential publication bias: This meta-analysis relied on publicly available literature, and positive outcomes are more likely to be published than negative or neutral ones. Besides, the quality of included studies varies, which may affect the stability of our conclusions. Only three prospective studies were included (16, 24, 33). Retrospective studies provide richer complication data, but incomplete datasets limit comprehensive analysis. Most studies report only one or a few complications. While our analysis increases sample size, it lacks long-term follow-up on fixed HSCT recipient populations. Such publication bias may have led to an overestimation or underestimation of the associations between conditioning regimens and outcomes.

Insufficient subgroup analyses for high-risk populations: Due to limited data availability in original studies, detailed subgroup analyses for high-risk CAYA subgroups (e.g., those with high-risk genetic mutations, early relapse, or aged <3 years) were not fully conducted. This limits the provision of targeted clinical recommendations for these specific populations.

Lack of standardized assessment of long-term complications: Different studies adopted varying diagnostic criteria and assessment tools for long-term complications (e.g., growth retardation, hypogonadism). The lack of standardization may have affected the accuracy and comparability of the pooled safety data.

Inability to account for confounding factors: Confounding variables such as prior treatment history, donor type, and post-transplant supportive care were not uniformly reported across original studies. These unadjusted factors may have influenced the comparative analysis of efficacy and safety between TBI and Bu-based regimens.

## Conclusion

In summary, among CAYA patients undergoing HSCT, busulfan-based conditioning confers survival benefits for those with AML and for cohorts comprising both malignant and non-malignant disease cases, whereas TBI showed advantages in EFS and relapse control for ALL patients. Regarding long-term adverse outcomes, five complications (growth retardation, thyroid dysfunction, hypogonadism, secondary tumors, and cataracts) occurred at significantly lower rates in the Bu group. Sex-related heterogeneity was noted for hypogonadism, where Bu exhibited a higher risk in the single female-only study. For cGVHD, the protective effect of Bu compared with TBI diminished as follow-up duration increased, although this trend did not reach statistical significance, as corroborated by meta-regression analysis. These findings highlight that physicians should individualize the choice of conditioning regimen for CAYA according to disease subtype. For ALL patients, the survival- and relapse-control advantages of TBI should be balanced against its elevated risk of long-term toxicities affecting children's growth, development and vital organ systems. For patients with AML, clinicians should take into account the survival benefits of Bu-based conditioning while remaining alert to potential subgroup-specific risks such as sex-dependent toxicity profiles. For CAYA receiving busulfan- or TBI-based conditioning, early preventive and interventional strategies, together with adequate education for patients and their guardians, are therefore clinically important.

## Data Availability

The original contributions presented in the study are included in the article/Supplementary Material, further inquiries can be directed to the corresponding author/s.
