# Supplementary material for: The complications of irradiation or busulfan for CAYA before hematopoietic stem cell transplantation: a systematic review and meta-analysis
Source: Front Pediatr. 2026 Sep 16;14:1865647. doi: 10.3389/fped.2026.1865647 (PMC13624081; doi:10.3389/fped.2026.1865647)
Supplement: Supplementary file 1 [file Supplementaryfile1.docx]

Linear regression test of funnel plot asymmetry:

1.OS

Test result: t = -0.00, df = 11, p-value = 0.9967. Bias estimate: -0.0107 (SE = 2.5028)

2.TRM

Test result: t = 1.38, df = 10, p-value = 0.1969. Bias estimate: 0.9008 (SE = 0.6515)

3.Relapse

Test result: t = 0.56, df = 9, p-value = 0.5921. Bias estimate: 0.7337 (SE = 1.3211)

4.cGVHD (OR)

Test result: t = 0.45, df = 8, p-value = 0.6672.

5.Others

Number of studies too small to test for small study effects (k.min=10).

Univariable study‑level meta‑regression:

1.OS (13 studies)

A. Study disease spectrum: The moderator accounted for 45.57% of the heterogeneity; the test for moderator was statistically significant (QM = 11.67, df = 3, P = 0.009). Using ALL‑only as reference group, logHR was significantly lower in AML‑only group and malignant and non‑malignant mixed group (P = 0.002 and P = 0.010, respectively), whereas no significant difference was found for mixed haematological malignancies group. Substantial residual heterogeneity remained after adjustment (residual I^2=91.17%, P<0.001).

2.EFS (9 studies)

A. Study disease spectrum: The moderator accounted for 72.96% of the heterogeneity; the test for moderator was statistically significant (QM = 19.70, df = 2, P < 0.001). Using ALL‑only as reference, logHR was significantly lower in AML‑only group and malignant and non‑malignant mixed group (P < 0.001 and P = 0.0052, respectively). Substantial residual heterogeneity remained after adjustment (residual I^2=86.01%, P<0.001). Given only 9 included studies, this meta‑regression result should be interpreted cautiously as exploratory.

3.TRM (12 studies)

A. Study disease spectrum: The moderator accounted for 0% of the heterogeneity; the test for moderator was not statistically significant (QM = 1.27, df = 3, P = 0.736). None of the comparisons against the ALL‑only reference group reached statistical significance. Residual heterogeneity was low (residual I^2=27.13%, P=0.169).

4.Relapse (11 studies)

A. Study disease spectrum: The moderator accounted for 51.38% of the heterogeneity; the test for moderator was statistically significant (QM = 7.20, df = 2, P = 0.027). Using ALL‑only as reference, significant difference was observed for AML‑only subgroup (P=0.007), whereas no significant difference was detected for mixed haematological malignancies group. Moderate residual heterogeneity was present after adjustment (residual I^2=43.44%, P=0.124).

5.cGVHD (OR) (10 studies)

A. Follow‑up duration: The moderator accounted for 99.98% of the heterogeneity; the test for moderator was not statistically significant (QM = 2.83, df = 1, P = 0.093). Using studies with follow‑up < 5 years as reference, the logOR difference for studies with follow‑up > 5 years was 0.46 (OR = 1.58, 95%CI: 0.93‑2.69, P = 0.093). Given only 10 included studies, this meta‑regression result should be interpreted cautiously as exploratory.

B. Study disease spectrum: The moderator accounted for 100.00% of heterogeneity; the test for moderators was not statistically significant (QM = 6.71, df = 4, P = 0.152). Using ALL‑only studies as reference, no statistically significant differences were observed for AML‑only, malignant‑non‑malignant mixed, MDS‑only, and mixed‑haematological‑malignancies subgroups (all P > 0.05). Given only 10 included studies, this meta‑regression result should be interpreted cautiously as exploratory.

5.Others (<10 sudies)

Given the limited number of included studies, univariable study‑level meta‑regression was not conducted owing to insufficient statistical power.

**Table 1.** Comparison of characteristics between TBI and busulfan groups of included studies and patients (comprehensively analyzed).

| **Study**  **(Author, year)** | **Bu** | | | | | **TBI** | | | | | **Outcomes** |
| --- | --- | --- | --- | --- | --- | --- | --- | --- | --- | --- | --- |
|  | **No. of patients** | **Age** | **Gender (male%)** | **Donor type** | **Disease** | **No. of patients** | **Age** | **Gender (male%)** | **Donor type** | **Disease** |  |
| **Bernard 2014** ^[15]^ | 66 | 6.7 (0.5–18.1) | 60.6 | Auto 28.8%  Allo 71.2% | AML 68.2%  ALL 31.8% | 174 | 8.43 (2.0–20.3) | 64.9 | Auto 23.6%  Allo 76.4% | ALL 86.8%  AML 13.2% | GR, TD, hypogonadism, ST, cataracts, NC, cGVHD |
| **Bunin 2003** ^[16]^ | 20 | NA (0.7-20) | 65 | Allo 100% | ALL 100% | 22 | NA (0.7-20) | 59.1 | Allo 100% | ALL 100% | cGVHD |
| **Cattoni 2022** ^[17]^ | 90 | NA | NA | Allo 100% | AML 64%  ALL 17%  Others 9%  JMML/CML 7%  Hemoglo-binopathy2%  SAA 1% | 108 | NA | NA | Allo 100% | ALL 89%  Lymphoma 7%  SAA 4% | TD, ST |
| **Cattoni 2023** ^[18]^ | 35 | NA (0-18) | 100 | Allo 100% | NA | 58 | NA  (0-18) | 100 | Allo 100% | NA | hypogonadism |
| **Cho 2011** ^[19]^ | 19 | NA | 0 | Allo  Auto | NA | 23 | NA | 0 | Allo  Auto | NA | hypogonadism |
| **Dandoy 2020** ^[48]^ | 425 | NA | 51 | Allo 100% | AML 100% | 199 | NA | 51 | Allo 100% | AML 100% | OS, EFS, TRM, relapse, cGVHD |
| **De Berranger 2014** ^[20]^ | 142 | 6.8 (0.5-17.9) | 45.1 | Allo 100% | AML 100% | 84 | 13.7 (2.6-17.9) | 50 | Allo 100% | AML 100% | OS, EFS, TRM, relapse, cGVHD |
| **Friend 2020** ^[21]^ | 30 | NA (3-25) | 57 | Allo 100% | ALL 100% | 27 | NA  (0-25) | 41 | Allo 100% | ALL 100% | OS, EFS, TRM, relapse, cGVHD |
| **Garming-Legert 2011** ^[22]^ | 25 | 7.7 (1-13) | 52 | Allo 100% | ALL, AML, CML, SAA, FHL, CGD, Metabolic disease | 49 | 7.7  (1-13) | 53.1 | Allo 100% | ALL, AML, MDS, CML, Lymphoma, SAA, FHL, CGD, Metabolic disease | cGVHD |
| **Hama 2023** ^[23]^ | 112 | NA (0-16) | NA | Allo  Auto | AML 100% | 65 | NA  (0-16) | NA | Allo  Auto | AML 100% | OS, EFS, TRM, relapse |
| **Horwitz 2015** ^[24]^ | 70 | NA | NA | Allo  Auto | AML, ALL | 201 | NA | NA | Allo  Auto | AML, ALL | cataracts |
| **Huttunen 2020** ^[25]^ | 11 | 6.3 (0.6–14.5) | 45 | Allo 100% | ALL 36%  AML/MDS 36%  CML 28% | 39 | 7.8 (2.7–17.1) | 67 | Allo 100% | ALL 64%  AML/MDS 13%  Nonmalignant 13%  JMML/CML 10% | OS, EFS, TRM, NC, cGVHD |
| **Ishida 2015** ^[26]^ | 69 | NA (0-17) | 55 | Allo 100% | AML 100% | 151 | NA  (0-17) | 46 | Allo 100% | AML 100% | OS, EFS, TRM, relapse, cGVHD |
| **Kato 2015** ^[27]^ | 90 | NA (3-12) | NA | Allo 100% | ALL 100% | 28 | NA  (3-12) | NA | Allo 100% | ALL 100% | OS, TRM, relapse, ST |
| **Keslova 2020** ^[28]^ | 175 | NA (0.1-20.5) | NA | Allo 100% | Malignancies  Non-malignancies | 146 | NA (0.1-20.5) | NA | Allo 100% | Malignancies  Non-malignancies | ST |
| **Liu 2022** ^[29]^ | 84 | NA (6-25) | 57.1 | Allo 100% | AML 69%  MDS  Others | 227 | NA  (6-25) | 56.8 | Allo 100% | ALL 69%  AML  MDS  Others | relapse, ST, cGVHD |
| **Lopez 2021** ^[30]^ | 53 | NA | 100 | Allo  Auto | AML  ALL | 178 | NA | 100 | Allo  Auto | AML  ALL | hypogonadism |
| **Lucchini 2017** ^[31]^ | 389 | 11.7 (2-18) | 53 | Allo 100% | AML 100% | 109 | 14.8 (2-18) | 58.7 | Allo 100% | AML 100% | OS, EFS, TRM, relapse, cGVHD |
| **Muñoz 2009** ^[32]^ | 17 | 8  (3-14) | 53 | Allo 100% | MDS 100% | 7 | 13  (7-18) | 29 | Allo 100% | MDS 100% | cGVHD |
| **Peters 2021** ^[33]^ | 102 | NA (4-21) | 61 | Allo 100% | ALL 100% | 201 | NA  (4-21) | 68 | Allo 100% | ALL 100% | OS, EFS, TRM, relapse, cGVHD |
| **Saglio 2020** ^[35]^ | 197 | 10  (3-18) | 61 | Allo 100% | AML 76%  ALL 24% | 473 | 9  (3-18) | 62 | Allo 100% | ALL 95%  AML 5% | OS, TRM, GR, TD, hypogonadism, ST, cataracts, cGVHD |
| **Styczynski 2004** ^[36]^ | 6 | 1.5 (0.7-14) | 50 | Allo 100% | Malignancies  Non-malignancies | 14 | 11 (2.1-18.5) | 64 | Allo 100% | Malignancies  Non-malignancies | OS, cGVHD |
| **Takahashi 2024** ^[37]^ | 370 | NA (0-21) | NA | Allo 100% | AML 100% | 180 | NA  (0-21) | NA | Allo 100% | AML 100% | OS, TRM, relapse, cGVHD |
| **Weinhard 2020** ^[38]^ | 38 | 5  (0-12) | 65.7 | Allo 100% | Non-malignancies 57.9%  AML 21%  ALL 5.3%  Other malignancies 15.8% | 36 | 9  (1-13) | 58.3 | Allo 100% | ALL 73.2%  AML 13.9%  Other malignancies  13.9% | hypogonadism |
| **Wilhelmson 2015** ^[39]^ | 30 | NA (0.4-19) | NA | Allo 100% | NA | 145 | NA (0.4-19) | NA | Allo 100% | NA | GR, TD, hypogonadism |
| **Yalcin 2021** ^[40]^ | 50 | 7.3 (0-18) | 58 | Allo 100% | ALL 100% | 22 | 9.4  (4-17) | 81.8 | Allo 100% | ALL 100% | OS, EFS, TRM, relapse, NC, cGVHD |

*Abbreviations: Bu, busulfan; TBI, total body irradiation; Auto, autologous; Allo, allogeneic; NA, not available; AML, acute myeloid leukemia; ALL, acute lymphoblastic leukemia; CML, chronic myelogenous leukemia; MDS, myelodysplastic syndromes; JMML, juvenile myelomonocytic leukemia; SAA, severe aplastic anemia; CGD, chronic granulomatous disease; FHL, familial hemophagocytic lymphohistiocytosis; OS, overall survival; EFS, event free survival; TRM, transplant-related mortality; GR, growth retardation; TD, thyroid dysfunction; ST, secondary tumors; NC, neurological complications; cGVHD, chronic graft-versus-host disease.

**Figure 1. Leave‑one‑out sensitivity analyses for survival endpoints.** A: Leave‑one‑out sensitivity analysis for overall survival (HR). B: Leave‑one‑out sensitivity analysis for event‑free survival (HR). C: Leave‑one‑out sensitivity analysis for transplant‑related mortality (HR). D: Leave‑one‑out sensitivity analysis for relapse (HR). Individual studies were iteratively excluded to test the stability of pooled hazard ratios.

Leave‑one‑out sensitivity analyses for survival endpoints showed that exclusion of any single study did not materially alter the pooled hazard‑ratio estimates, suggesting robust results.

**Figure 2. Funnel plots for late‑onset complications to evaluate publication bias.** A: Funnel plot for cataracts (OR). B: Funnel plot for cataracts (HR). C: Funnel plot for cGVHD (OR). D: Funnel plot for cGVHD (HR). E: Funnel plot for second tumour (OR). F: Funnel plot for thyroid dysfunction (OR). G: Funnel plot for growth retardation (OR). H: Funnel plot for neurological complications (OR). I: Funnel plot for hypogonadism (OR). Asymmetry of funnel plots implies potential publication bias.

Funnel plots for cGVHD (OR), secondary tumor, and hypogonadism showed mild visual asymmetry, which may hint at potential publication bias or small‑study effects. Given the small number of studies per endpoint, formal asymmetry tests were not conducted.

**Figure 3. Subgroup analyses of secondary outcomes (neurological complications, growth retardation, cGVHD) of patients included in the meta-analysis.** A: Forest plot of subgroup analyses of neurological complications stratified by median follow-up (OR). B: Forest plot of subgroup analyses of neurological complications stratified by disease spectrum (OR). C: Forest plot of subgroup analyses of growth retardation stratified by transplant setting (OR). D: Forest plot of subgroup analyses of cGVHD stratified by disease spectrum (OR). E: Forest plot of subgroup analyses of cGVHD stratified by disease spectrum (HR). F: Forest plot of subgroup analyses of growth retardation stratified by disease spectrum (OR). G: Forest plot of subgroup analyses of neurological complications stratified by transplant setting (OR).

**Figure 4. Subgroup analyses of secondary outcomes (hypogonadism, thyroid dysfunction, second tumor) of patients included in the meta-analysis.** A: Forest plot of subgroup analyses of hypogonadism stratified by disease spectrum (OR). B: Forest plot of subgroup analyses of hypogonadism stratified by transplant setting (OR). C: Forest plot of subgroup analyses of thyroid dysfunction stratified by disease spectrum (OR). D: Forest plot of subgroup analyses of thyroid dysfunction stratified by transplant setting (OR). E: Forest plot of subgroup analyses of second tumor stratified by disease spectrum (OR). F: Forest plot of subgroup analyses of second tumor stratified by transplant setting (OR). G: Forest plot of subgroup analyses of second tumor stratified by median follow-up (OR).
